# Risky decision-making following prefrontal D1 receptor manipulation

**DOI:** 10.1515/tnsci-2020-0187

**Published:** 2021-11-03

**Authors:** Dominik K. E. Beyer, Lisa Horn, Nadine Klinker, Nadja Freund

**Affiliations:** Division of Experimental and Molecular Psychiatry, Department of Psychiatry, Psychotherapy and Preventive Medicine, LWL University Hospital, Ruhr-University, 44801 Bochum, Germany

**Keywords:** Iowa gambling task, lentivirus, rat, bipolar disorder, mania

## Abstract

The prefrontal dopamine D1 receptor (D1R) is involved in cognitive processes. Viral overexpression of this receptor in rats further increases the reward-related behaviors and even its termination induces anhedonia and helplessness. In this study, we investigated the risky decision-making during D1R overexpression and its termination. Rats conducted the rodent version of the Iowa gambling task daily. In addition, the methyl CpG–binding protein-2 (MeCP2), one regulator connecting the dopaminergic system, cognitive processes, and mood-related behavior, was investigated after completion of the behavioral tasks. D1R overexpressing subjects exhibited maladaptive risky decision-making and risky decisions returned to control levels following termination of D1R overexpression; however, after termination, animals earned less reward compared to control subjects. In this phase, MeCP2-positive cells were elevated in the right amygdala. Our results extend the previously reported behavioral changes in the D1R-manipulated animal model to increased risk-taking and revealed differential MeCP2 expression adding further evidence for a bipolar disorder-like phenotype of this model.

## 1 Introduction

The dopaminergic system has pleiotropic effects on the brain and is involved in motivation, reward, hedonia, cognitive function, and risk-taking behavior [1]. Viral overexpression of the dopamine D1 receptor (D1R) in the medial prefrontal cortex (mPFC) of adult rats was associated with increased drug seeking and taking, impulsivity, hedonic, and sexual behavior [2,3]. Interestingly, when terminating the overexpression, animals show anhedonia, hypoactivity, increased anxiety, and helplessness indicating a switch from mania- to depressive-like behavior [2,4]. In healthy controls, too much or too little D1R stimulation in the PFC has a negative effect on cognitive processes [5]. Furthermore, D1R density within the mPFC is increased following 60 min training of working memory in mice [6]. However, the effect of prefrontal D1R overexpression and its termination on cognitive processes remains unclear.

One aspect of cognition is decision-making, the process of making a specific choice between various alternatives. Hereby the alternatives can vary in risk and/or amount of a specific outcome. One possibility to access decision-making in humans is the Iowa gambling task (IGT) [7,8]. In short, the participants investigate and evaluate several options trial by trial to maximize their outcome. They choose between different decks. Some decks are associated with a higher immediate gain but are overall disadvantageous due to their higher chance of higher penalties. Advantageous decisions are associated with smaller immediate gain, but a smaller chance for penalties. The performance of Parkinson’s patients, a disorder associated with degeneration of dopaminergic neurons, within the IGT is influenced by dopaminergic medication [9]. Moreover, dopamine dysregulation is associated with mood disorders, especially bipolar disorder (BD), and major depressive disorder [10,11]. In addition to cycling in hedonia, locomotor and sexual activity, and anxiety, BD patients exhibit neurocognitive impairments. Cognitive dysfunctions are present during all illness phases, even in euthymia [12,13]. Decision-making of BD patients in the IGT is often shifted towards more risky choices or disadvantageous decisions, whereby decision-making seems to be independent of the current episode [14,15,16,17]. These findings in the IGT reflect elevated risk-taking behavior and impulsivity, which are omnipresent in the daily life of BD patients like excessive drug consumption, risky driving, and sexual activities [18,19,20]. In addition, the risk to develop gambling problems is several times higher in BD patients compared to the general population [21]. BD and gambling disorders have one thing in common: dysregulation of the dopaminergic system [11,22,23]. The dopaminergic system can influence the performance in the IGT. Reducing dopamine levels in healthy controls induced a shifting towards disadvantageous decisions in the IGT [24] and the release of dopamine in the ventral striatum correlates with the performance within the IGT in healthy controls and pathological gamblers, but in an opposite direction [25]. In addition, dopamine is crucial to weigh a prediction and adapt decisions depending on reward contingencies [26].

In rodents, an adapted version of the IGT, the so called rat gambling task (RGT) has been used [27]. The RGT based on the same principle as that of the IGT, investigates the same core features, and therefore crucially contributes to the scientific field of decision-making [28]. Manipulation of the dopaminergic system in animals highlighted the important role of dopamine in the RGT. Dopamine transporter knockdown mice make more risky or disadvantageous decisions in the RGT [15,29]. One potential molecular mechanism for risky decision-making is methyl CpG–binding protein-2 (MeCP2) [30]. MeCP2 is a key regulator of gene transcription and has therefore several downstream targets [31]. Neurotransmitter systems associated with psychiatric disorders, such as GABA, dopamine, and dopamine receptors, are influenced via MeCP2 manipulation [32,33,34]. Deletion of MeCP2 induces, among other things, a reduction in dopamine within the striatum [33]. The striatum is important for decision-making [35,36,37]. In addition, performance in the risky decision-making task (RDT) led to changes in MeCP2 expression throughout the brain. MeCP2 expression was reduced in the basolateral amygdala (BLA), nucleus accumbens, and dorsal mPFC in rats preferring risky decision-making directly after the RDT. In addition, MeCP2 expression in the mPFC is inversely correlated with risky choices even 7 days after the RDT, highlighting the possibility of rapid changes in MeCP2 expression. Furthermore, phosphorylation of MeCP2 was increased with a preference for risky decision-making [30]. MeCP2 plays a key role in gene transcription and is associated with dopamine dysregulation [32,33,34]. Patients with type II BD show increased levels of MeCP2 mRNA in peripheral blood cells [38]. The previously observed high-risk behavior following manipulation of the prefrontal D1R [3] may as well depend on differential MeCP2 expression.

The goal of the present study was to investigate risky decision-making in the D1R-manipulated rat model and to identify if MeCP2 expression is altered. The RGT is based on operant conditioning and can be evaluated daily. Therefore, decision-making could be observed during D1R overexpression and after the termination within one animal.

## 2 Material and methods

### 2.1 Animals

A total of 13 adult, male Sprague Dawley rats were obtained from Charles River Laboratories (Sulzfeld, Germany). Rats were pair-housed in a standard cage with water available *ad libitum* in constant temperature and humidity conditions (22 ± 2°C and 55 ± 25%) on an inverted 12 h light/dark cycle (light on at 11 p.m.). Behavioral measures began no earlier than 1 h after the beginning of the dark phase of the cycle. One week before behavioral procedures, the animals were allowed to acclimate to the animal facility. During behavioral procedures, rats were food restricted to 35–40 g/cage, available after each training and testing session. Animal weight was controlled daily to maintain their weight at constant level.


**Ethical approval:** The research related to animals’ use has been complied with all the relevant national regulations and institutional policies for the care and use of animals.

### 2.2 Lentiviral vector

#### 2.2.1 Virus production

The used lentiviral construct was described previously [2]. Briefly, a Tetracycline-On inducible lentiviral vector system (Tet.On) was used, coding for the rat D1R protein (*DRD1* gene) or for control condition for the red fluorescent protein dsRed. A calmodulin Kinase II subunit alpha (CamKIIα) promoter resulted in constitutive regulation of rtTA3 expression and dsRed or D1R expression was regulated by the tetracycline derivative doxycycline (DOX). Virus production was performed by the Viral Core Facility, Charité Universitätsmedizin Berlin based on the protocol of Stewart and colleagues [39] with the use of plasmids 8,454 and 8,455 by Addgene.

#### 2.2.2 Surgery

20 min before surgery rats received metamizole (100 mg/kg), were anesthetized with isoflurane, and 1 µL of virus (2 × 10^7^ transducing units per µL) per hemisphere was bilaterally injected into the mPFC at stereotaxic coordinates AP: + 2.7, ML: ±0.4, DV: −2.8 according to the rat brain atlas [40].

#### 2.2.3 DOX treatment

Virus expression was activated via 0.5 g/l DOX (Sigma-Aldrich) administration in the drinking water to produce an “ON” state. Animals received 7 continuous days of DOX (“ON” state) followed by 7 days of DOX withdrawal (“OFF” state).

### 2.3 Behavioral procedures

#### 2.3.1 Behavioral setup

Behavioral testing took place in an operant chamber (30 cm × 30 cm × 30 cm). Operant chamber was controlled by a Raspberry Pi 3B + connected to a touchscreen and feeder to deliver sugar pellets automatically. Custom RGT training and testing programs were written in MatLab (Mathworks; Natick, MA) in combination with the Biopsychology-Toolbox [41].

#### 2.3.2 RGT training

Prior to surgery, rats learned to touch a wide white strip on the touchscreen to receive a reward. In the next training session, subjects were trained to associate three continuous hits on the wide strip to be rewarded with a sugar pellet. Rats had an answer time of 10 s to interact with the touchscreen, followed by an inter-trial interval (ITI) of 10 s. Each animal was trained for 60 min each day for at least 5 days in each training phase. The last training block consisted of the training of the actual RGT. RGT test procedures were adapted from previously established protocols [27]. Two consecutive choices on the same symbol were needed to be counted as a directed and voluntary choice and was rewarded with food. The rats could freely choose between four symbols, which were presented at the same spatial location and are associated with different reward and penalty (a time-out) probabilities. The overall advantageous and disadvantageous decisions are described in detail in Table 1.

**Table 1:** Summary of parameters used for assessing decision-making and risk-taking behavior via the RGT

Symbol	Reward (pellets)	Penalty duration (s)	Penalty probability (%)	Theoretical maximum benefit (pellets)	Overall decisions
A	2	222	50	28	Disadvantageous
B	2	444	25	28	Disadvantageous
C	1	12	25	156	Advantageous
D	1	6	50	156	Advantageous

#### 2.3.3 RGT test

**Figure 1:**
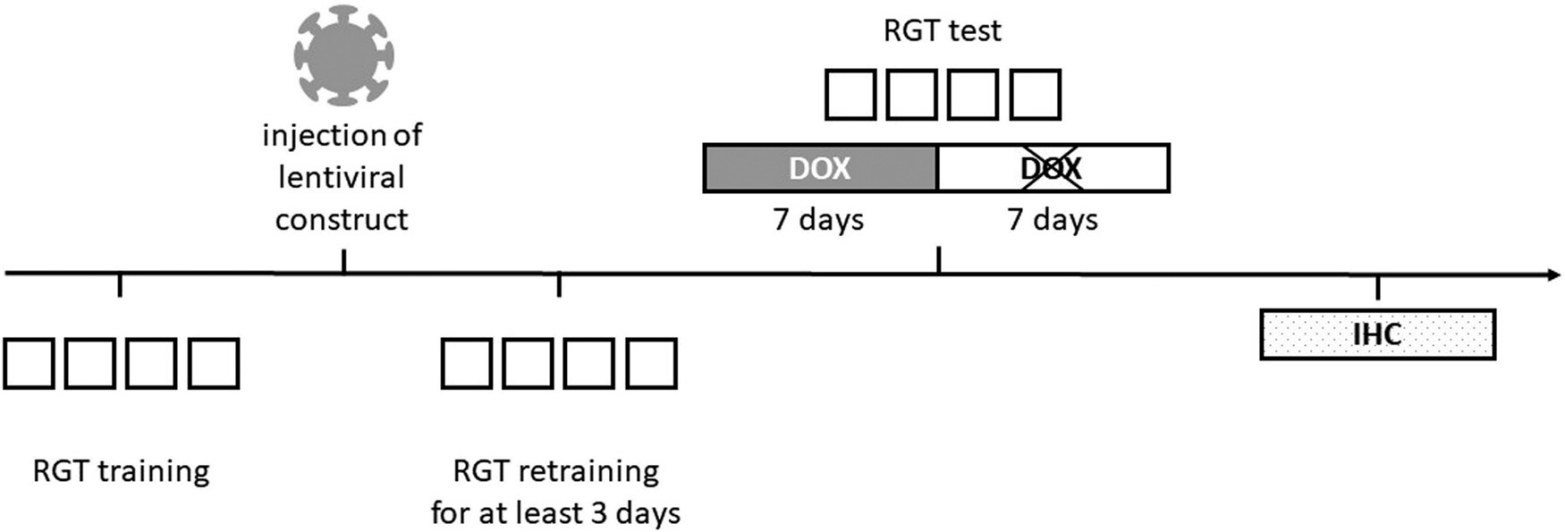
Experimental design. At first, rats performed the RGT training procedure. Afterward, glutamatergic neurons within the mPFC were transduced with D1R or dsRed. Then RGT retraining for at least 3 days took place. The decision-making was daily accessed throughout 7 days of viral overexpression of D1R or dsRed induced via doxycycline (DOX) administration. Furthermore, DOX was removed and viral overexpression terminated the following 7 days in which decision-making was further accessed daily. Subjects were sacrificed one day after the last RGT test.

Surgery was performed after the subjects successfully learned the RGT task. One animal had to be excluded because it did not learn the RGT task. Therefore, the final sample size amounted to six animals for each group. After surgery, the animals were allowed a recovery phase of 3 days without RGT testing. Afterwards, the rats performed the RGT paradigm for 3 continuous days, referred to as baseline measurement, before DOX treatment started, and the assessment of risky decision-making behavior took place over a period of 14 days (Figure 1).

The test consisted of a single 1 h session each day. Subjects had free access to the previously learned symbols (star, quadrat, triangle, and circle), but each choice was associated with different consequences, which resulted in an overall advantageous or disadvantageous decision-making (Table 1). Choices A and B resulted in an immediate reward of two pellets, whereas C and D resulted in only one pellet. Overall, choices A and B are disadvantageous although two pellets were delivered, because this bigger reward could be followed by very high penalties (time-outs of 222 or 444 s). In comparison, choices C and D were advantageous because the smaller reward was followed by smaller penalties (time-outs of 6 and 12 s). Penalty probabilities were high for A and D (50%) and low for B and C (25%). During the penalty, the rats had to wait in front of a black screen to continue with the task to earn more reward. The presentation of all symbols signaled the end of the time-out and allowed the animal to go on with the task. Trials associated with no penalty had no time-out. Each trial was separated by an ITI of 10 s. The theoretical maximum benefit was the same for choices A and B and for choices C and D. Overall, choices C and D would theoretically allow gaining 5.6 times more pellets than choices A and B. With this design, risky decision-making could be assessed in a longitudinal manner. In addition, it was investigated whether subjects choose an advantageous or disadvantageous choice following a long penalty. The selection of an advantageous symbol is referred to as safe and the selection of a disadvantageous symbol is referred to as risky choice.

### 2.4 Immunohistochemistry (IHC), cell counting, and analysis of MeCP2 staining

Rats were deeply anesthetized with a ketamine/xylazine mixture (100/10 mg/kg) and intracardially perfused with 4% paraformaldehyde as previously described [42]. Brains were cryoprotected and cut into 40 µm coronal sections. To identify MeCP2-positive cells, a previously established protocol was used [43] with slight modifications. In short, sections were permeabilized with 0.03% PBST for 60 min at room temperature and then blocked for 60 min at room temperature with 16% normal goat serum and exposed overnight at 4°C to 1:1,000 mouse anti-MeCP2 (Abcam). Next day, the sections were washed in 1× PBS, and incubated for 60 min at room temperature with 1:1,500 anti-mouse Alexa 488-coupled IgG (Sigma-Aldrich). The sections were again washed in 1× PBS, incubated with 20 µg/mL 4′,6-diamidino-2-phenylindole (DAPI), washed, and mounted on slides. Images of each hemisphere for each region of interest (mPFC: bregma + 2.7 and BLA: bregma + 2.04) were generated with a fluorescence microscope (Axio Imager M1) and the software Zen (Zeiss) under consistent exposure time. MeCP2- and DAPI-positive cells were counted separately for each hemisphere by an investigator blinded in terms of experimental conditions via ImageJ [44].

### 2.5 Virus placement

Virus placement was verified via PCR after behavioral testing. Genomic DNA was isolated from mPFC sections via QIAamp DNA FFPE Tissue Kit (Qiagen). The following primers were used to evaluate the virus placement against rtTA3 with the previously purified DNA: forward primer 5′GGAGGAACAGGAGCATCAAG3′ and reverse primer 5′GGCAGCATATCAAGGTCAAAG3′. 10× PCR-Buffer (MgCl_2_), 25 mM MgCl_2_, 10 mM dNTPs, Taq-Polymerase, and 70 ng DNA were filled up to a final volume of 52.8 µL with H_2_O. The PCR protocol consisted of an initiation denaturation at 95°C for 5 min, followed by 50 cycles at 95°C for 30 s, annealing for 30 s at 60°C, and primer extension for 1 min at 72°C, and a final extension at 72°C for 10 min followed by holding at 4°C in the cycler (Eppendorf). All amplified results were analyzed via agarose gel electrophoresis, staining with Midori Green (NIPPON Genetics), and visualization of DNA bands using an UV transilluminator (Vilber). The synthesized amplicon for the rtTA3 had a size of 264 bp and determined by DNA Ladder (100 bp, New England Biolabs). Only subjects with a positive detection of the rtTA3 PCR product were used for data analysis.

### 2.6 Statistics

SPSS statistical software 26 was used to perform statistics. Kolmogorov–Smirnov test was used to check for normal distribution of data. Dependent variables (behavioral data) were analyzed with repeated measures ANOVA with three factors: RGT testing day, virus group (D1R and dsRed), and virus state (“ON” and “OFF”). IHC of MeCP2-positive and DAPI-positive cells were analyzed for each region via ANOVA with virus group, region (mPFC and BLA), and hemisphere (left and right) as between factors. Correlation between MeCP2 immunoreactivity and behavior was assessed with Pearson correlation coefficients. In all cases, *p* < 0.05 was considered statistically significant.

## 3 Results

### 3.1 RGT performance is affected by prefrontal D1R manipulation

All animals were included for data analysis due to a positive verification of viral placement. Manipulation of the mPFC via overexpression of D1R or dsRed and its termination had no effect on the general ability to perform the task. The number of responses during the 14 days of RGT testing did not differ between the groups (testing day X virus group interaction, F(1.7, 16.5) = 1.4, *p* = 0.27, testing day X virus group X virus state interaction, F(2.5, 25.5) = 1.6, *p* = 0.2).

**Figure 2:**
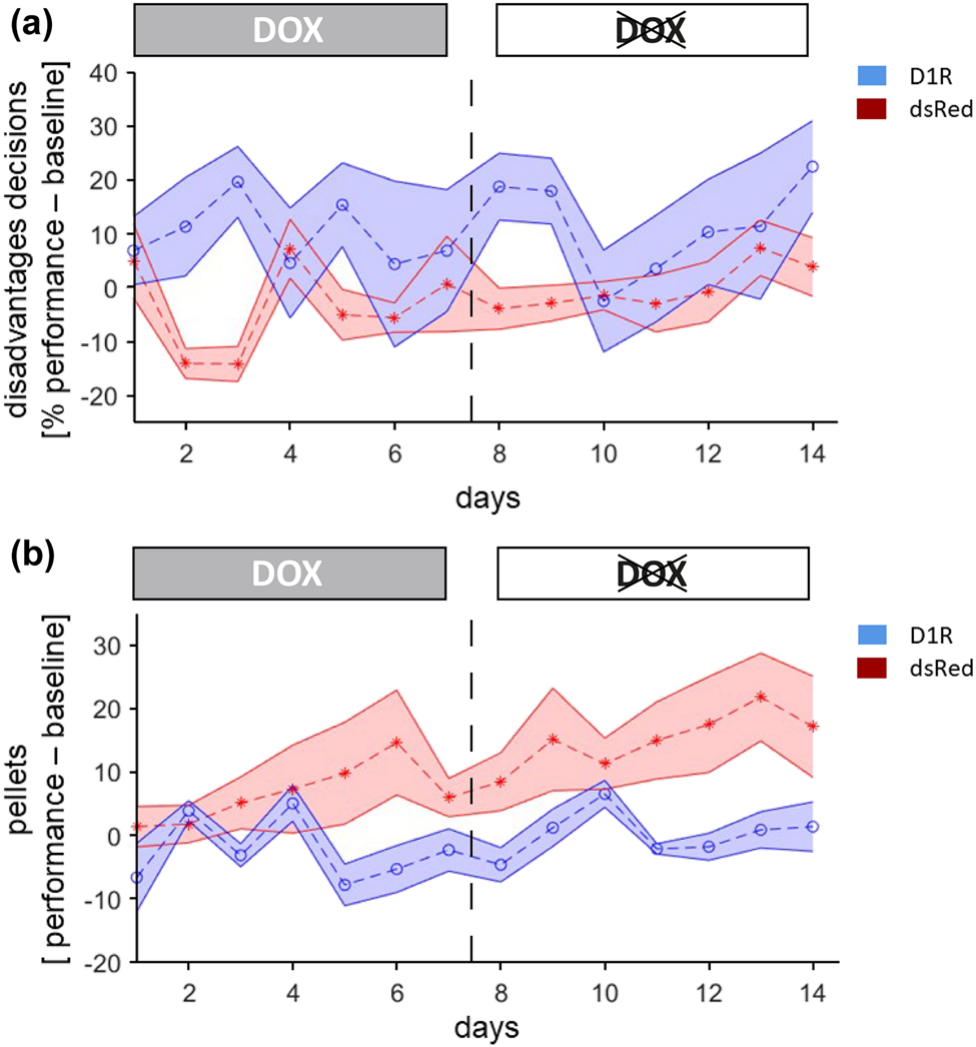
Assessment of risky decision-making and learning during both episodes in the animal model for BD. Disadvantageous decision-making is delineated as percentage of performance – baseline and anhedonia/learning as earned pellets (performance – baseline). (a) Risky decision-making is significantly affected by D1R over-expression. Termination of viral overexpression results in a normalization of decision-making in D1R subjects. (b) Learning is not affected during D1R overexpression. Termination of D1R overexpression results in learning deficits although no alteration in decision-making was observable.

Baseline measurements of decision-making in the RGT took place after surgery and prior DOX treatment. Surgery had no significant effect on disadvantageous decisions during baseline measurements (testing day X virus group interaction, F(2, 20) = 0.6, *p* = 0.5). Therefore, baseline measurement could be used to account for interindividual differences between single subjects and calculate the number of disadvantageous decisions during testing condition in relation to baseline by subtracting the number of disadvantageous decisions for each test day from the mean number of disadvantageous decisions during baseline. However, raw data is illustrated in Table 2. During testing, a significant interaction of testing day X virus group X virus state (F(6, 60) = 2.79, *p* = 0.018) was found. To determine at what virus state the risky decision-making was affected, both virus states (“ON” and “OFF”) were investigated separately (Figure 2). During the “ON” state, when overexpression of D1R occurred, a significant testing day X virus group interaction (F(6, 60) = 2.4, *p* = 0.038) was found. In the “OFF” state after termination of viral overexpression, risky decision-making did not significantly differ (testing day X virus group interaction, F(2.6, 26.8) = 1.5, *p* = 0.25). Statistical analysis testing day for each testing day revealed that D1R subjects choose more disadvantageous decisions compared to dsRed animals on day 3, day 8, and day 9 (uncorrected students *t*-test, *p* < 0.05). Individual stimuli were analyzed to determine if D1R manipulation influenced a specific pattern of choice. No significant interaction of testing day X virus group X virus state was found for symbol A (F(6, 60) = 0.6, *p* = 0.7), B (F(6, 60) = 0.9, *p* = 0.5), C (F(6, 60) = 1.7, *p* = 0.13), or D (F(6, 60) = 0.6, *p* = 0.7). To further investigate the pattern of decision-making, the responses to a long delay were investigated. Manipulation had no significant effect on number of safe choices (testing day X virus group X virus state interaction, F(3.6, 36.3) = 0.6, *p* = 0.6) or risky choices (testing day X virus group X virus state interaction, F(2.7, 26.6) = 1.4, *p* = 0.27) following a long penalty.

**Table 2:** Disadvantageous decisions in the RGT throughout viral overexpression and its termination. Means and standard errors are listed

Baseline							
Days	−2	−1	0				
D1R	51 ± 10	41 ± 14	52 ± 13				
dsRed	51 ± 17	41 ± 10	54 ± 16				
“ON” state							
Days	1	2	3	4	5	6	7
D1R	56 ± 12	60 ± 14	68 ± 10	53 ± 14	64 ± 9	53 ± 11	56 ± 11
dsRed	47 ± 17	29 ± 12	29 ± 12	49 ± 17	38 ± 15	37 ± 15	43 ± 18
“OFF” state							
Days	8	9	10	11	12	13	14
D1R	67 ± 8	67 ± 10	46 ± 11	52 ± 12	59 ± 11	60 ± 15	70 ± 12
dsRed	38 ± 15	39 ± 14	41 ± 14	39 ± 16	42 ± 17	49 ± 17	46 ± 17

In addition, the amount of earned pellets was investigated throughout the experiment. Baseline measurements revealed that no significant interaction of testing day X virus group (F(2, 20) = 0.1, *p* = 0.9) was present prior to DOX treatment. During testing, no significant interaction of testing day X virus group X virus state (F(2.9, 28.9) = 1, *p* = 0.4) was found. However, DOX treatment and therefore virus state significantly affected the amount of earned pellets (F(1, 10) = 7.8, *p* = 0.019). But this effect is only observable in dsRed subjects (F(1, 5) = 6.3, *p* = 0.05) and not in D1R animals (F(1, 5) = 1.5, *p* = 0.27), with dsRed animals earning more pellets during the OFF phase compared to the ON phase (t(12) = −3.6, *p* = 0.003).

### 3.2 Termination of prefrontal D1R overexpression affects MeCP2

**Figure 3:**
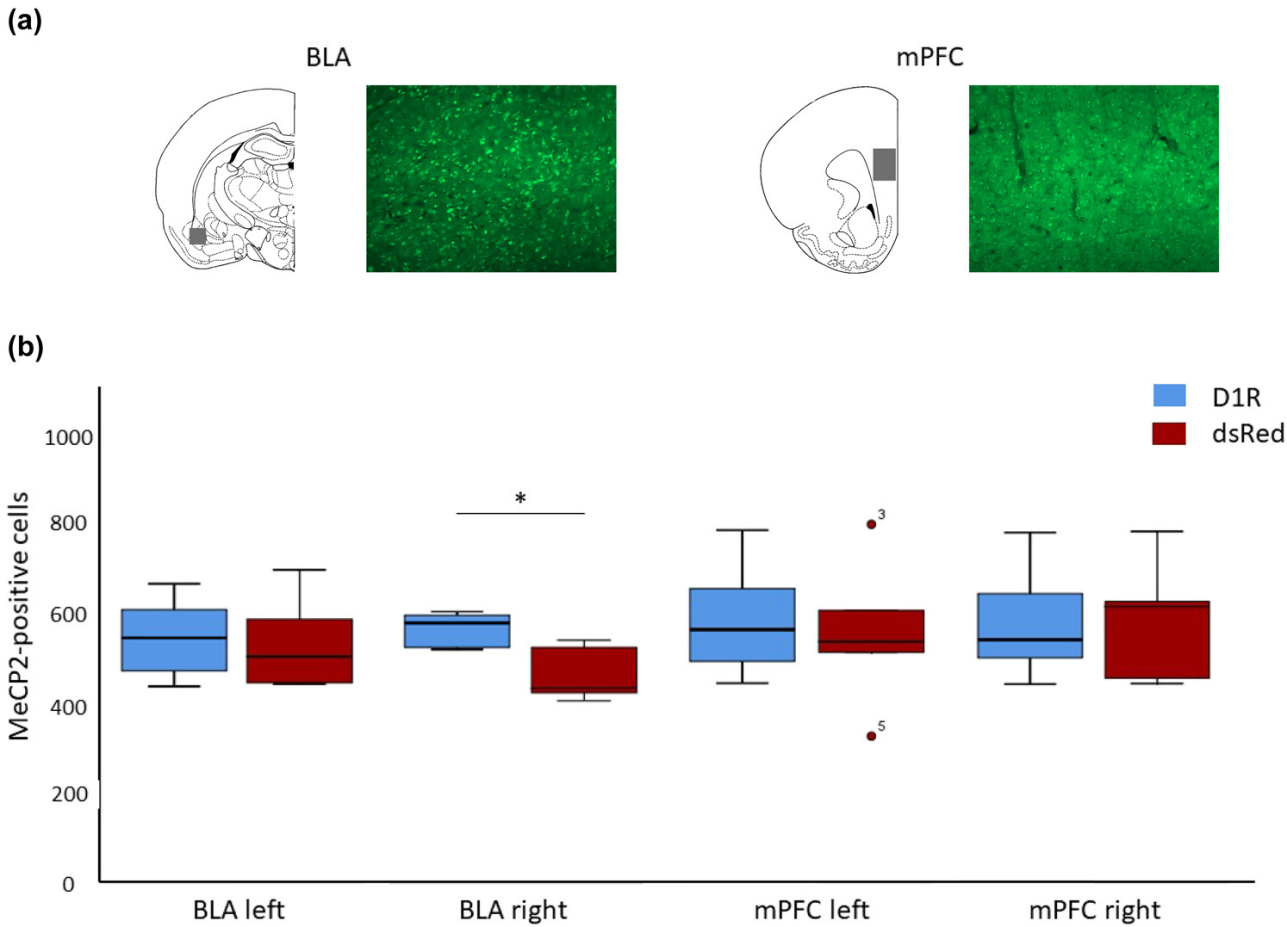
Immunohistochemical stainings. MeCP2-positive cells following viral overexpression and its termination in the left and right hemispheres of the BLA and mPFC. (a) The amount of MeCP2-positive cells in the right BLA is significantly increased in D1R “OFF” subjects compared to controls (b).

D1R manipulation had a significant virus group X brain region X hemisphere interaction (F(1, 10) = 10.9, *p* = 0.008). Therefore, the brain region and hemispheres were investigated separately in the following part. D1R manipulation did not affect the amount of MeCP2-positive cells in the whole mPFC (F(2, 9) = 0.4, *p* = 0.7), neither in the left (F(1, 10) = 0.1, *p* = 0.7) nor right hemispheres (F(1, 10) = 0.06, *p* = 0.8) (Figure 3). However, termination of D1R viral overexpression had an effect on MeCP2-positive cells in the whole BLA (F(2, 9) = 7.3, *p* = 0.013). This effect is exclusively driven by changes in the amount of MeCP2-positive cells in the right (F(1, 10) = 14.8, *p* = 0.003) and not the left hemisphere (F(1, 10) = 0.09, *p* = 0.7). *Post-hoc* comparison revealed that the number of MeCP2-positive cells was significantly increased in the whole BLA, especially the right BLA in D1R subjects compared to dsRed animals (Bonferroni correction, *p* < 0.05).

To determine if changes in MeCP2-positive cells are dependent on total cell numbers, DAPI-positive cells were analyzed. D1R manipulation did not affect the amount of DAPI-positive cells within the whole BLA (F(2, 9) = 1.5, *p* = 0.28), nor left (F(1, 10) = 0.2, *p* = 0.6) or right hemispheres (F(1, 10) = 1.7, *p* = 0.2) of the BLA.

### 3.3 Correlation between MeCP2 immunoreactivity and risky decision-making

To further analyze if MeCP2 and decision-making are connected, a correlation between disadvantageous decisions and MeCP2-positive cells was performed. Analysis revealed a trend for a connection between the elevated number of MeCP2-positive cells in the mPFC and less disadvantageous decisions (*r* = −0.54, *p* = 0.07). Furthermore, the amount of earned pellets and MeCP2 expression were analyzed. Indeed, analysis revealed a strong trend for an association between the number of earned pellets and MeCP2 expression in the right BLA (*r* = −0.57, *p* = 0.057).

## 4 Discussion

Our results demonstrate risky decision-making in the RGT following D1R overexpression in the mPFC. D1R “ON” subjects choose more disadvantageous decisions compared to dsRed “ON” animals. The term disadvantageous decisions is based on the assumption that the outcome over a long period of time or many decisions return less earnable reward. In addition, disadvantageous choices are associated with a high-risk high-reward pattern. Here the observed maladaptive behavior is in line with mania-like behavior following optogenetic stimulation of D1R [45] and D1R overexpression within the mPFC [2,3]. D1R levels are age-dependent, increase rapidly in the mPFC during development, peak in adolescence, and decrease in adulthood [46,47]. The D1R peak in the mPFC goes along with increased risk-taking in adolescence. This connection is no coincidence as D1R signaling furthermore contributes to effort-based decision-making [48] and altered D1R levels can lead to maladaptive decision-making. The increased risk-taking and maladaptive decision-making in D1R “ON” subjects is an indicator for mania-like behavior. The same behavioral pattern can be observed in BD patients within a manic episode. Manic patients tend to choose more risky choices and engage in risky activities with a high potential for negative consequences [14,15,16,17,49]. Furthermore, mania is associated with elevated impulsivity and a general risk-taking behavior, which can be accessed via several clinical tests [50,51,52]. The prevalence of BD patients with gambling problems is several times higher compared to the general population [21]. One neurobiological overlap of BD patients [11] and gambling addiction [22,23] is a dysregulation of the dopamine system. In addition, BD patients exhibit altered levels of D1R [53,54,55].

Interestingly, the increase in risky decision-making started as early as 3 days after the administration of DOX and lasted (with irregularities in between) until 2 days after DOX removal. The observed risky decision-making in D1R subjects beyond day 7, the last day of DOX administration, might be due to properties of the inducible lentiviral construct. The Tet.On system needs *in vivo* 3 days of DOX withdrawal till the expression of the gene of interest is turned off [56]. It was previously shown that it takes 3 days after DOX removal till D1R animals exhibit a switch from mania- to depressive-like behavior [2]. The same time pattern for a behavioral switch is observable in the presented data.

Irregularities, namely similar decision-making of both groups on day 4 to day 7 might be due to inter-individual differences and intra-individual differences between days. Risky decision-making increased from day 3. This specific amount of time is necessary until viral constructs with a Tet.On system change the morphology of the magnitude of cells [57] or lead to measurable differences in the organism of rodents [58].

After termination of viral overexpression, no differences in risky decision-making could be observed between D1R transduced subjects and controls. However, while the number of earned pellets over time increased in control animals, this effect could not be observed in D1R manipulated animals. As in the DOX “OFF” phase, neither risky decisions nor number of completed trials differed between groups, it cannot be interpreted as a general increase in motivation or decrease in risky decisions in the control group, but a combination of both. D1R-manipulated animals in contrast did not increase the number of earned pellets. Previous studies reported depressive-like behavior of D1R “OFF” subjects indicated via increased anxiety, learned helplessness, reduced sucrose preference, and decreased locomotor activity [2,4]. The reduced amount of earned pellets in the D1R “OFF” animals could result from improved learning of dsRed subjects, which was not observable in D1R rats. We are not able to distinguish if less earned reward reflects learning deficits or anhedonia in D1R “OFF” subjects. However, anhedonia was not measured directly, although the lack of interest in sugar pellets could indicate anhedonia based on previous studies. Nonetheless, impaired learning as well as anhedonia are characteristic features of BD patients in a depressive episode [12].

The role of prefrontal D1R in depression is in line with the fact that the antidepressant effect of levo-stepholidine depends on its function as an D1R agonist within the mPFC [59].

One potential mechanism connecting the dopamine manipulation, risky decision-making, and BD is MeCP2 and its key role in affecting gene transcription. After 7 days of DOX administration followed by 7 days of DOX withdrawal, D1R-manipulated animals showed an elevated number of MeCP2-positive cells in the right BLA. No differences in the total cell numbers were found between groups indicating that altered number of MeCeP2-positive cells are not based on overall cell quantity differences. Interestingly, during this phase, no differences in risky decision-making were observed between groups. Deng et al. (2018) observed a decreased MeCP2 expression in the BLA in animals that had performed a decision-making task compared to animals that had not performed.

It has been previously reported that manipulation of MeCP2 influences neurotransmitter systems, such as GABA [60], dopamine, and dopamine receptors, especially dopamine D2 receptor, in several brain regions which are associated with psychiatric disorders [33,34]. In addition, *Mecp2*-null mice display reduced dopamine within the striatum [33]. Here we give a first indication that manipulation of D1R was able to influence MeCP2 expression. Even though the D1R manipulation was conducted in both hemispheres of the mPFC, MeCP2 was mainly affected in the right BLA. Abnormalities of the amygdala are associated with BD patients. The bilateral amygdala is enlarged in BD patients compared to healthy controls [61,62] and a higher number of BD episodes correlate with larger amygdala volumes [63]. Even the current episode can influence amygdala connectivity. Manic patients displayed a decreased connectivity in the amygdala [64]. Interestingly, depressed and hypomanic BD patients exhibit increased connectivity, especially in the right amygdala [65]. Amygdala connectivity in BD is even sensitive to reward, such as sparse connectivity for anticipated loss or denser connectivity for win anticipation compared to healthy controls [66]. Hemispheric asymmetries are also present in BD patients [67]. Several studies found a higher activation of the right hemisphere compared to the left hemisphere in manic patients, whereas depressed patients displayed an opposite effect [68,69]. This asymmetry can be observed in the amygdala and is accompanied with an increased activity of the right amygdala in depressed BD patients [70]. Those findings are in line with differences in MeCP2-positive cells in the right hemispheric BLA of D1R “OFF” subjects.

In the present study, a potential new animal model for BD was further characterized and risky decision-making was investigated in a longitudinal manner. Increased risk-taking during the proposed mania-like phase could be reduced to baseline with the induced switch to the depressive-like phase. However, the standard deviation was high due to a low number of animals per group. Therefore, an increase and decrease in risky decisions over time were not clearly detectable. Using operant conditioning but with a less complex task might result in a more pronounced switch. An additional limiting factor of the study is the fact that MeCP2 was investigated when no difference in risky decision-making was observed between the groups. During the “ON” phase, additional differences in MeCP2 expression comparable to that reported by Deng et al. might have been observed. Another limitation is that only males were involved in this study. Differential D1R signaling between sexes may contribute to sex specific vulnerability to consequences of social withdrawal, anxiety-, and depressive-like behaviors [71,72].

## 5 Conclusion

In conclusion, D1R overexpression within the mPFC-induced maladaptive risky decision-making is an indication for mania-like behavior. Termination of viral overexpression resulted in return to control the levels of risky decision-making, but at the same time reduced motivation to earn reward was also observed. The previously found bipolar-like behavior after D1R manipulation in the mPFC [2] was confirmed and extended to risky decision-making in the “ON” (mania-like) state. Interestingly, neurobiological mechanisms are even altered in the “OFF” state which could explain the switch to not only baseline but to depressive-like behavior. The protein MeCP2, which affects gene expression, is increased in the BLA of D1R “OFF” subjects in an asymmetric manner with a stronger effect in the right BLA.

## List of abbreviations

BD: bipolar disorder
BLA: basolateral amygdala
CaMKII: calmodulin kinase II
DAPI: 4′,6-diamidino-2-phenylindole
DOX: doxycycline
D1R: dopamine D1 receptor
IGT: iowa gambling task
IHC: immunohistochemistry
ANOVA: analysis of variance
MeCP2: methyl CpG–binding protein-2
mPFC: medial PFC
RDT: risky decision-making task
RGT: rat gambling task
Tet.On: tetracycline-on inducible lentiviral vector system
